# Protective efficacy induced by *Eimeria maxima* rhomboid-like protein 1 against homologous infection

**DOI:** 10.3389/fvets.2022.1049551

**Published:** 2023-01-04

**Authors:** Mingyue Wang, Di Tian, Lixin Xu, Mingmin Lu, Ruofeng Yan, Xiangrui Li, Xiaokai Song

**Affiliations:** MOE Joint International Research Laboratory of Animal Health and Food Safety, College of Veterinary Medicine, Nanjing Agricultural University, Nanjing, China

**Keywords:** *Eimeria maxima*, ROM1, DNA vaccine, subunit vaccine, protective efficacy

## Abstract

**Introduction:**

Avian coccidiosis, caused by apicomplexan protozoa belonging to the *Eimeria* genus, is considered one of the most important diseases in the intensive poultry industry worldwide. Due to the shortcomings of live anticoccidial vaccines and drugs, the development of novel anticoccidial vaccines is increasingly urgent.

**Methods:**

*Eimeria maxima* rhomboid-like protein 1 (EmROM1), an invasion-related molecule, was selected as a candidate antigen to evaluate its protective efficacy against *E. maxima* in chickens. Firstly, the prokaryotic recombinant plasmid pET-32a-EmROM1 was constructed to prepare EmROM1 recombinant protein (rEmROM1), which was used as a subunit vaccine. The eukaryotic recombinant plasmid pVAX1.0-EmROM1 (pEmROM1) was constructed as a DNA vaccine. Subsequently, 2-week-old chicks were separately vaccinated with the rEmROM1 and pEmROM1 twice every 7 days. One week post the booster vaccination, induced cellular immune responses were determined by evaluating the mRNA level of cytokines including IL-2, IFN-γ, IL-4, IL-10, TGF-β, IL-17, and TNFSF15, as well as the percentages of CD4^+^ and CD8^+^ T cells from spleens of vaccinated chickens. Specific serum antibody level in the vaccinated chickens was determined to assess induced humoral immune responses. Finally, the protective efficacy of EmROM1 was evaluated by a vaccination-challenge trial.

**Results:**

EmROM1 vaccination significantly upregulated the cytokine transcription levels and CD4^+^/CD8^+^ T cell percentages in vaccinated chickens compared with control groups, and also significantly increased the levels of serum-specific antibodies in vaccinated chickens. The animal trial showed that EmROM1 vaccination significantly reduced oocyst shedding, enteric lesions, and weight loss of infected birds compared with the controls. The anticoccidial index (ACI) from the rEmROM-vaccination group and pEmROM1-vaccination group were 174.11 and 163.37, respectively, showing moderate protection against *E. maxima* infection.

**Discussion:**

EmROM1 is an effective candidate antigen for developing DNA or subunit vaccines against avian coccidiosis.

## 1. Introduction

Chicken coccidiosis is an intracellular parasitic protozoa disease caused by *Eimeria* coccidia and leads to devastating financial losses worldwide (1). Blake et al. (2) recently estimated the economic losses caused by chicken coccidiosis at £10.4 billion globally in 2016. In the United States, chicken coccidiosis (especially *E. maxima*) was ranked first among the major disease-related issue in the broiler industry in 2019 and 2020 surveys by the United States Animal Health Association (3). In the United Kingdom, chicken coccidiosis was ranked in the top three major avian diseases, given its economic significance (2). In China, chicken coccidiosis has recently been listed as a Class 3 animal disease, according to the Ministry of Agriculture and Rural Affairs Administration (4). The causative pathogens of chicken coccidiosis were reported to be seven apicomplexan species belonging to the *Eimeria* genus (5). Among all chicken *Eimeria* species, *E. maxima* is one of the major pathogenic species found in intensive chicken farms (6). For instance, the infection rate of *E. maxima* was reported to be 73% in an epidemiological survey of *Eimeria* species carried out by Williams et al. in France (7). Thebo et al. reported that the infection rate of *E. maxima* was the highest among the seven *Eimeria* species, reaching 63.4% in Sweden (8). McDougald et al. (9) found an infection rate was 42% for *E. maxima*, close to that of *E. tenella* in an epidemiological survey conducted in Argentina. Bachaya et al. investigated the prevalence of coccidiosis in southern Punjab, Pakistan. They found that the occurrence rate of chicken coccidia was 65%, among which the infection rate of *E. maxima* was 31.38%, slightly lower than that of *E. tenella* (10). Lan et al. sampled five large chicken farms in Zhejiang province, China, and found that the infection rate of *E. maxima* was ranked third (21.1%) across other *Eimeria* spp. (11). As a result, the timely prevention and control of *E. maxima* are of vital importance for the sustainable development of the poultry industry.

At present, the control of chicken coccidiosis mainly depends on the usage of anticoccidial drugs and vaccination (12). With the increasing attention of the World Health Organization to food safety and health, the frequent use of drugs will lead to the resistance of species, and the safety issues of animal-derived food caused by drug residues will also be the Achilles heel for the prevention and control of chicken coccidiosis (13). Immunoprophylaxis is a promising method to reduce or replace the use of anticoccidial drugs. Currently, available commercial anticoccidial vaccines are mainly live vaccines including virulent vaccines (CocciVac-B, CocciVacD, and Immucox, etc.) and attenuated vaccines (Paracox and Livacox, etc.) (12). However, the use of live vaccines has complicated production processes, high production expenses, and an atavistic possibility of coccidian, which suggests that the importance of research on alternative methods. The first commercialized subunit vaccine against coccidiosis is CoxAbic, which is composed of purified native proteins isolated from the gametocytes of *E. maxima*. The vaccine works through a strategy of maternal immunization, which provides protection against coccidiosis of the offspring chickens through vaccination of laying hens (13, 14). However, its application is limited due to the difficulties associated with antigen production and the complicated immunization program (12).

Recently, it has been suggested that next-generation recombinant anticoccidial vaccines are novel methods for controlling coccidiosis because of their unique advantages, such as safety, low cost, convenient mass production, and encouraging protection efficacy. A couple of potential vaccine antigens have been identified to develop next-generation vaccines. These candidates, in their forms of plasmid DNA, recombinant protein, or live-vectored formulations, exhibited promising protections against coccidiosis in reducing oocyst outputs and body weight loss and alleviating enteric lesions (5, 15, 16). The rhomboid-like proteins (ROMs) have been reported to play various essential roles in multiple organisms. For example, they were involved in shedding adhesins from the surface of serval apicomplexan protozoa during the invasion of host cells (17–19). The invasion-related roles of ROMs indicate that they could be used as candidate antigens to develop novel vaccines for controlling avian coccidiosis (20–23). In the present study, we evaluated the immune responses and protective efficacy against *E. maxima* challenge infection induced by EmROM1 in its forms of plasmid DNA (pEmROM1) and recombinant protein (rEmROM1). This study provided a promising candidate antigen to develop the next-generation recombinant vaccines against avian coccidiosis.

## 2. Materials and methods

### 2.1. Parasite, chickens, and vectors

*E. maxima* were isolated, purified, and preserved in our laboratory. Fresh sporulated oocysts were prepared by propagating through chickens seven days before the challenge experiment. Newly hatched chicks (Hy-Line) were raised in wire cages and supplied with sufficient feed and water without anticoccidial drugs. SD (Sprague-Dawley) rats were bought from Qinglongshan Animal Breeding Farm in Nanjing. *E. coli* DH5α and *E. coli* BL21 were purchased from Vazyme (Nanjing, China). The prokaryotic expression vector of pET-32a(+) was from Novagen (Darmstadt, Germany), and the eukaryotic expression vector of pVAX1.0 was from Invitrogen (Carlsbad, CA, USA).

### 2.2. Cloning, expression, and plasmid construction of EmROM1 gene

The micro glass beads (0.1–0.2 mm; Omega Bio-Tek, Norcross, United States) were used to break the oocysts, and the breaking rate of sporulated oocysts was up to 80% (24). The total RNA of sporulated *E. maxima* oocysts was extracted by E.Z.N.A. TM Total RNA Kit I (Omega Bio-Tek, Norcross, United States). Then a reverse transcription assay was carried out to produce the cDNA of the parasite with the total RNA as a template using a Reverse Transcription Kit (Vazyme, Nanjing, China). Subsequently, a PCR assay was conducted to generate the EmROM1 segments for the construction of pET-32a-EmROM1 and pVAX1.0-EmROM1 recombinant vectors. Specific primers #1, namely *Bam*H I anchored forward primer (5′-CGGATCCATGCCTGTCTGCACACT-3′) and *Hin*d III anchored reverse primer (5′-CCAAGCTTTTATG CACTGCATCCCCTAT-3′) were used for constructing pET-32a-EmROM1. Specific primers #2, namely *Bam*H I anchored forward primer (5′-CGCGGATCCATGCCTGTCTGCACACT-3′) and *Eco*R I anchored reverse primer (5′-CCGGAATT CTTATGCACTGCATCCCCTAT-3′), were used to construct pVAX1.0-EmROM1. The PCR procedure was set as follows, an initial denaturation at 94°C for 5 min, followed by 35 cycles at 94°C for 30 s, 60°C for 30 s, and 72°C for 52 s. Finally, the PCR product was ligated with pET-32a(+) and pVAX1.0, producing recombinant pET-32a-EmROM1 and pVAX1.0-EmROM1 vectors, which were then transformed into *E. coli* BL21 and DH5α, respectively. Endonuclease digestion and sequencing analysis were conducted to verify the newly reconstructed plasmids.

### 2.3. Purification of recombinant protein EmROM1 (rEmROM1) and preparation of polyclonal antibody against rEmROM1

To obtain rEmROM1, pET-32a-EmROM1 transformed bacteria were cultured until the bacterial solution OD 600 reached the range of 0.6–0.8. Then IPTG induction was carried out to promote the expression of rEmROM1 with a working concentration of 1 mM. A His TrapTM FF crude kit (5 mL; GE Healthcare, Piscataway, United States) was used to purify the rEmROM1.

To prepare polyclonal antibody against rEmROM1, SD rats were injected through subcutaneous immunization in the back with 250 μg rEmROM1 plus Freund's Adjuvant Complete (FCA, Sigma-Aldrich, St. Louis, United States). Two weeks after the primary immunization, the rats were immunized with the same dose of rEmROM1 antigens emulsified in Freund's Adjuvant Incomplete (FICA, Sigma-Aldrich, St. Louis, United States) once a week. After the fifth immunization, blood samples were obtained from the orbital veins. Indirect ELISA was carried out to detect the antiserum titer and stored at −70°C. In addition, the antiserum of naïve and pET-32a tag protein was obtained by referring to the above method, respectively as controls.

### 2.4. Recognition of rEmROM1 by anti-*E. maxima* chicken serum

The purified rEmROM1 was transferred from the SDS-PAGE gel to the nitrocellulose membrane. After transfer, the membrane was washed in PBST (phosphate buffer saline containing 0.05% Tween-20) 3 times. After washing, the membrane was blocked in 5% BSA at 4°C overnight. The membrane was incubated with the primary antibody of anti-*E. maxima* chicken serum (1:100 diluted by 5% BSA) for 4 h at 25°C. Meanwhile, uninfected chicken serum was used as a naïve control. The membrane was washed with PBST 3 times after the primary antibody incubation. Then, the membrane was incubated in HRP-conjugated Goat anti-chicken IgG (1:4,500 diluted by 5% BSA; Biodragon, Beijing, China) at 37°C for 1.5 h in the dark. After 3-time washing, the bound antibody was detected with a DAB kit in the dark.

### 2.5. Detection of transcription and expression of pEmROM1 in chickens

Two-week-old healthy chickens were divided into two groups randomly. One group was injected with 100 μg of pEmROM1 through intramuscular immunization in the legs, and the other was immunized with 100 μg of pVAX1.0 as negative vector control. After 7 days of immunization, the muscles were obtained and cut into pieces with sterile scissors from the injection site and the non-injection site, respectively. Subsequently, muscle RNA was extracted by RNAiso Plus kit (TaKaRa, Kyoto, Japan) following the product instruction. The gDNA Wiper Mix (Vazyme, Nanjing, China) was used to remove the residual DNA or plasmid. Then reverse transcription was conducted to produce cDNA. After that, the PCR procedure with the specific primers was carried out to analyze the transcription of EmROM1 at the injection site and the non-injection site. Meanwhile, the muscles were lysed with RIPA solution for 2 h and centrifuged to obtain the supernatant at 10,000 g for 10 min for Western blot analysis with rat anti-rEmROM1 serum (1:100 diluted by 5% BSA) as primary antibody and HRP-conjugated Goat anti-chicken IgG (1:4,500 diluted by 5% BSA) as the second antibody. Unimmunized rat serum was used as a naïve control.

### 2.6. Detection of the rEmROM1-induced immune response in chickens

#### 2.6.1. Animal immunizations and sample collections

To detect rEmROM1-induced immune response, 14-day-old chickens were randomly divided into 3 groups of 30 chickens per group. The experimental group was immunized with 200 μg of rEmROM1 in the leg muscles of chickens. The control groups were immunized with 200 μg of pET-32a tag protein and 200 μL of PBS, respectively. At the age of 21 days, a booster injection was given with the same protocol of primary immunization. On the 7th day after the primary immunization and the booster immunization, 5 chickens were selected randomly to isolate spleen lymphocytes to analyze T cell subsets and cytokines expression. Meanwhile, chicken serum was collected to detect the specific antibody level.

The same experimental regime was used to detect the pEmROM1-induced immune response. The experimental group was immunized with 100 μg of pEmROM1, and the control groups were immunized with 100 μg of pVAX1.0 vector and 100 μL of PBS, respectively.

#### 2.6.2. Detection of the T Lymphocyte Subsets from the vaccinated chickens by Flow cytometry

Spleen lymphocytes were distributed into 2 tubes with 10^7^ lymphocytes in each tube. One was double-stained with 2 μL of Mouse Anti-chicken CD3 (Southern Biotechnology Associates, Birmingham, United States) and 2 μL of Mouse Anti-chicken CD4 (Southern Biotechnology Associates, Birmingham, United States), and the other was double-stained with 2 μL of Mouse Anti-chicken CD3 and 2 μL of Mouse Anti-chicken CD8 (Southern Biotechnology Associates, Birmingham, United States). For the PBS control group, the lymphocytes were divided into one with non-staining, one with 2 μL of CD3 single staining, and the other with 2 μL of CD4 or CD8 single staining. All were incubated at 4°C for 45 min in the dark. After that, the cells were washed with PBS and centrifuged at 500 g for 3 min. The lymphocyte precipitation was gently blown with PBS and transferred into the flow tube. The lymphocytes were detected with a FACS Calibur flow cytometer (Becton Dickinson, New York, United States). The PBS control group of lymphocytes, including the non-staining and CD4 or CD8 single staining, were used as template regulation. Flow cytometry data were processed and exported using the FlowJo V10 (Becton Dickinson, New York, United States).

#### 2.6.3. Detection of the mRNA level of spleen cytokines from vaccinated chickens by quantitative real-time PCR (qPCR)

NCBI and Primer Ques Tool (IDT; Bethesd, Maryland, United States) was used to design cytokines primers for qPCR (IL-2, IL-4, IL-10, IL-17, IFN-γ, TGF-β, and TNF SF15), GAPDH as an internal control. RNA was extracted by RNA Extraction Kit from isolated spleen lymphocytes and reversely transcribed into cDNA as a template. The reaction contained 10 μL of 2 × ChamQ SYBR qPCR Master Mix (Vazyme, Nanjing, China), 2 μL of cDNA, 0.4 μL of primer F, 0.4 μL of primer R, 0.4 μL of 50 × ROX Reference Dye 2 (Vazyme, Nanjing, China) and 6.8 μL of RNase-Free water that was measured in triplicate. Specific primers with amplification efficiency ranging from 90 to 110% and ΔCq ≧ 3 were screened by gradient-diluted cDNA. Cytokines were measured with specific primers, and mRNA levels of cytokines were calculated by 2^−Δ*ΔCt*^. The data of qPCR was processed and exported using the 7500 Software v 2.3 (Applied Biosystems, Foster City, United States).

#### 2.6.4. Detection of antibody level of IgG from immunized chicken serum by ELISA

Blood collection from immunized chickens after the primary immunization, with 6 consecutive collections at an interval of 1 week. The blood samples were cultured at 37°C for 1.5 h, and then put at 4°C for 8 h. Serum was obtained to detect IgG antibody levels after centrifuging at 1,000 g for 8 min. ELISA was conducted to detect IgG from chicken serum. Briefly, a 96-well microtiter plate was coated with the rEmROM1 or pET-32a tag protein (1.5 μg per well) at 4°C overnight. After 5-time washing with PBST, the plates were blocked with 4.5% skimmed milk at 25°C for 5 h. After 3-time washing, chicken serum (1:100 dilution) was used as the primary antibody, and added to the incubated plate (100 μL per well) at 25°C for 3 h. Meanwhile, a negative control was added with non-immunized diluted chicken serum (1:100 dilution), and the blank control was added with 100 μL PBS. After 5-time washing with PBST, 100 μL of Goat anti-chicken IgG (1: 4,500 dilution, Biodragon, Beijing, China) was added as the secondary antibody at 25°C for 2.5 h. After 3-time washing, the plate was incubated with TMB (3,3,5,5-tetramethylbenzidine) at 25°C for 8 min in the dark. Finally, the assay was terminated and measured at 450 nm (OD_450_) by spectrophotometer (Thermo Fisher Scientific, Waltham, United States).

### 2.7. Evaluation of protective efficacy induced by EmROM1 against *E. maxima*

#### 2.7.1. Animal grouping and immunization

Two immunization-challenge trials were performed to evaluate the efficacy induced by EmROM1 against *E. maxima* infection. The protective efficacy induced by rEmROM1 against *E. maxima* was evaluated in trial 1. Fourteen-day-old chickens were randomly assigned into 4 groups (30 per group), including the experimental group, pET-32a tag protein, challenged, and unchallenged groups. The experimental group was given 200 μg of rEmROM1 twice by intermuscular immunization per 7 days. The pET-32a tag protein group was given pET-32a tag protein. The challenged and unchallenged groups were given sterile PBS.

The protective efficacy induced by pEmROM1 was evaluated by trial 2 against *E. maxima*. Fourteen-day-old chickens were randomly assigned into 4 groups (30 per group), including the experimental group, pVAX1.0 vector, challenged, and unchallenged groups. The experimental group was given 100 μg pEmROM1 twice by the same method as the rEmROM1 immunization. The pVAX1.0 vector control was immunized with pVAX1.0 plasmid. The challenged, and unchallenged control groups were immunized with sterile PBS.

#### 2.7.2. Protective efficacy evaluation

Seven days post the booster immunizations, chickens were orally challenged with 1 × 10^5^ of *E. maxima* sporulated oocysts per chicken except for the unchallenged group. Six days post the challenge infection, chickens were slaughtered. The protective efficacy of EmROM1 was evaluated by recording weight gain, oocyst output, and enteric lesion score and calculating the anticoccidial index (ACI) (25–29). The weight gain was calculated with the following formula: body weight at the end of the experiment—that at the challenge. The survival rate was calculated by the number of surviving chickens dividing that of the initial chickens. The oocysts of per gram feces (OPG) and enteric lesion score were determined (27, 30). ACI was calculated: (relative rate of weight gain + survival rate)–(lesion index + oocyst index) (31).

### 2.8. Statistical analysis

All data were analyzed by SPSS 20 statistical analysis software, and significant differences between groups were analyzed using one-way ANOVA. *P* > 0.05 was set as non-significant. *P* < 0.05 was set as significant.

## 3. Results

### 3.1. Cloning, expression, and plasmid construction of EmROM1 gene

**Figure 1:**
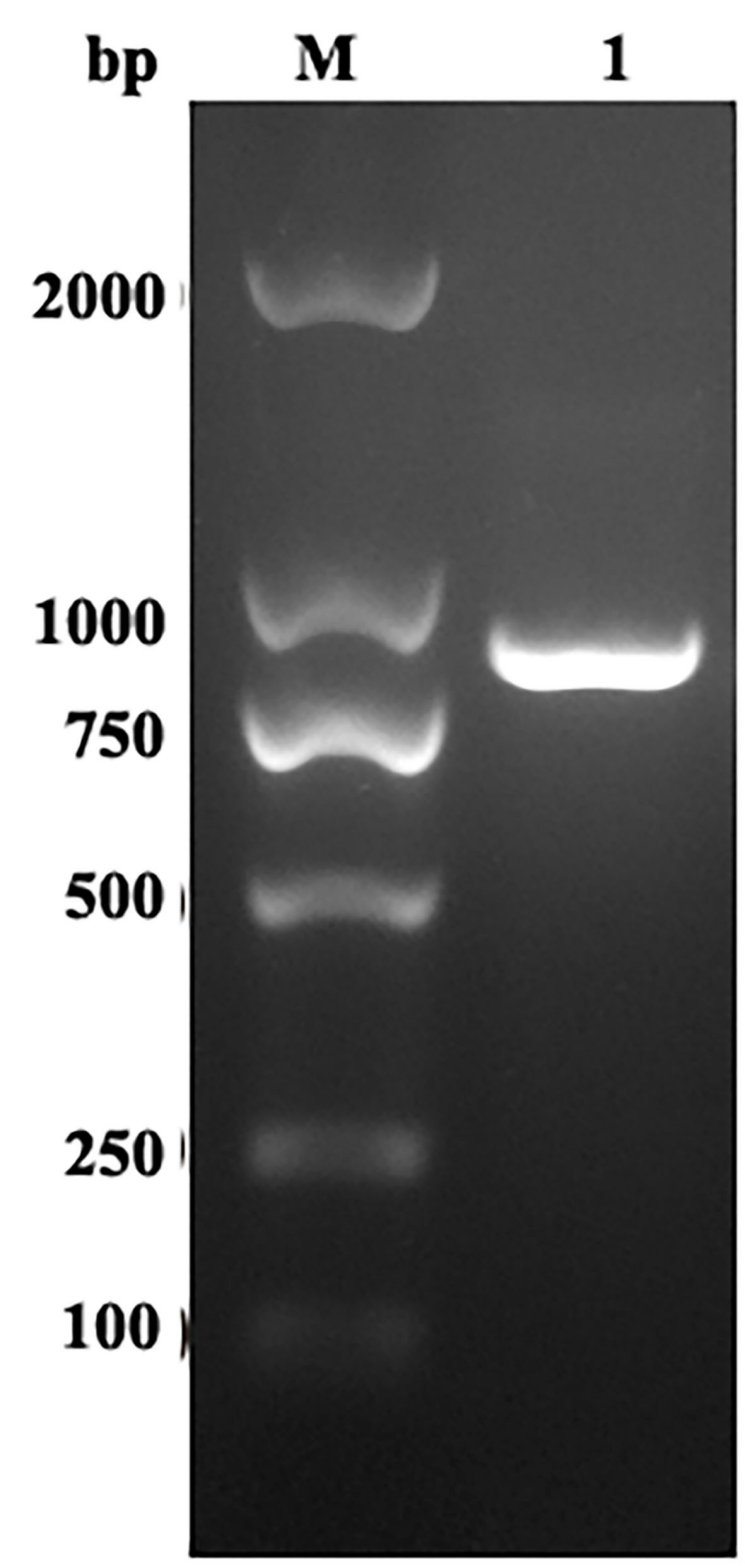
RT-PCR amplification of EmROM1. M: DNA Molecular Weight Standard DL2000. Lane 1: Amplification products of EmROM1.

The EmROM1 gene was cloned from *E. maxima* by reverse-transcription PCR. Agarose gel electrophoresis revealed a band of about 888 bp (Figure 1), which was consistent with the size of ORF of EmROM1. The ORF of EmROM1 was constructed into pET-32a and pVAX 1.0, respectively, to generate pET-32a-EmROM1 and pVAX1.0-EmROM1 recombinant plasmids. The band of about 888 bp was examined from digested pET-32a-EmROM1 vectors *via* double enzyme digestion (*Bam*H I and *Hin*d III). Similarly, a band of about 888 bp was examined from pVAX1.0-EmROM1 digested with *Bam*H I and *Eco*R I. The constructed recombinant plasmids were further verified by sequencing. The results showed that the ORF of EmROM1 was 888 bp, sharing a similarity of 100% to the target EmROM1 sequence posted in GenBank (sequence ID: XM_013479531.1).

### 3.2. Expression, purification, and western blot analysis of rEmROM1

**Figure 2:**
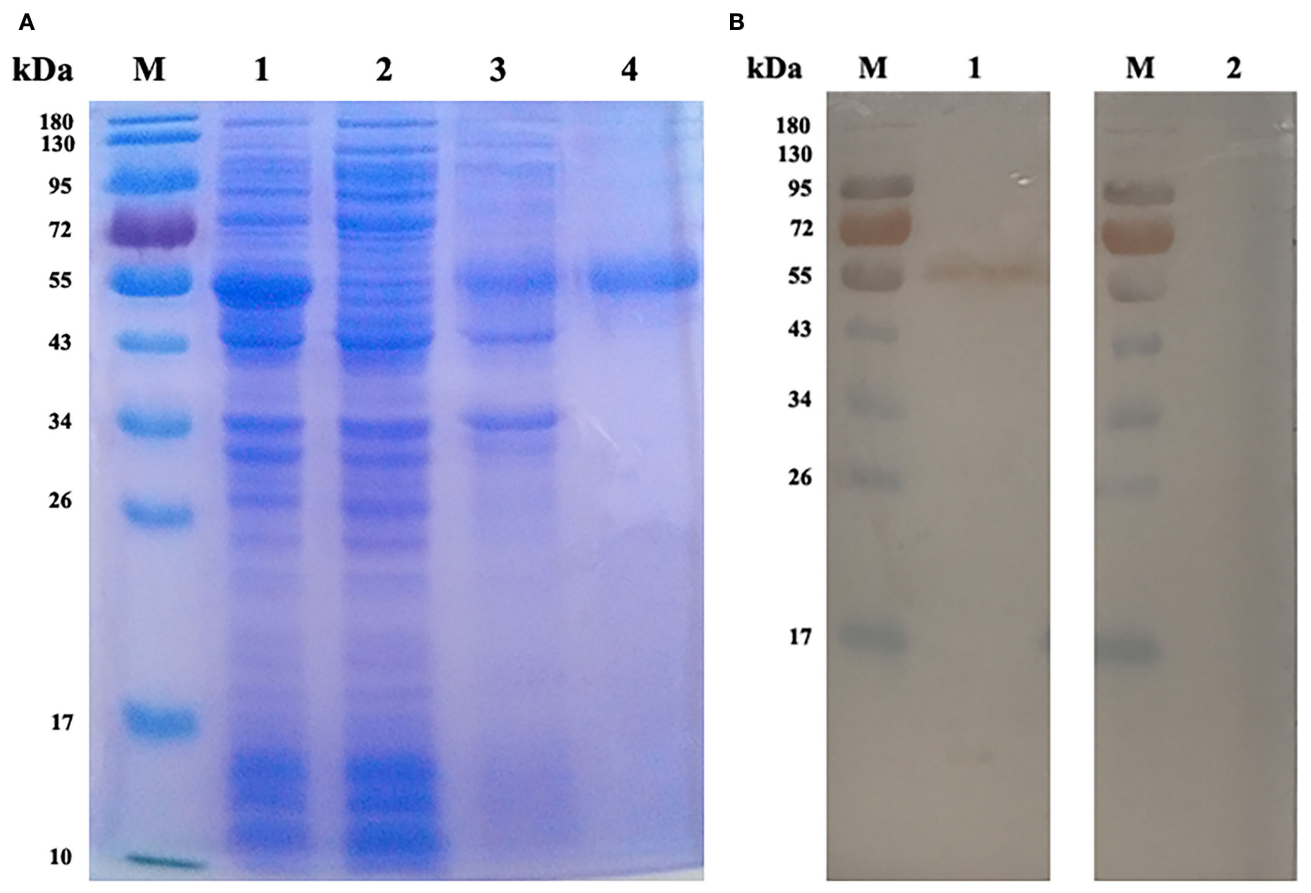
Expression and Western blot analysis of rEmROM1. **(A)** Expression and purification of rEmROM1. M: protein standard molecular weight. Lane 1: pET-32a-EmROM1-transfected bacteria induced by IPTG for 5 h. Lane 2: Cell lysate supernatant of pET-32a-EmROM1 bacterial liquid induced by IPTG for 5 h. Lane 3: Cell lysate sediment of pET-32a-EmROM1 bacterial liquid. Lane 4: rEmROM1 after purification. **(B)** Western blot analysis of rEmROM1. M: protein standard molecular weight. Lane 1: Identification of rEmROM1 by *E. maxima* infected chicken serum. Lane 2: Recognition of rEmROM1 by negative chicken serum.

The rEmROM1 with His tag was induced in *E. coli* BL21 (DE3) and purified by Ni-NTA affinity chromatogram. The SDS-PAGE gel characterized a single band of about 50.56 kDa (Figure 2A), which was basically consistent with the total molecular weight of rEmROM1 (about 32.5 kDa) and pET-32a tag protein (about 18 kDa). Western blot determined that rEmROM1 was recognized by anti-*E. maxima* chicken serum (Figure 2B).

### 3.3. Transcription and expression of pEmROM1 at the injection site in chickens

**Figure 3:**
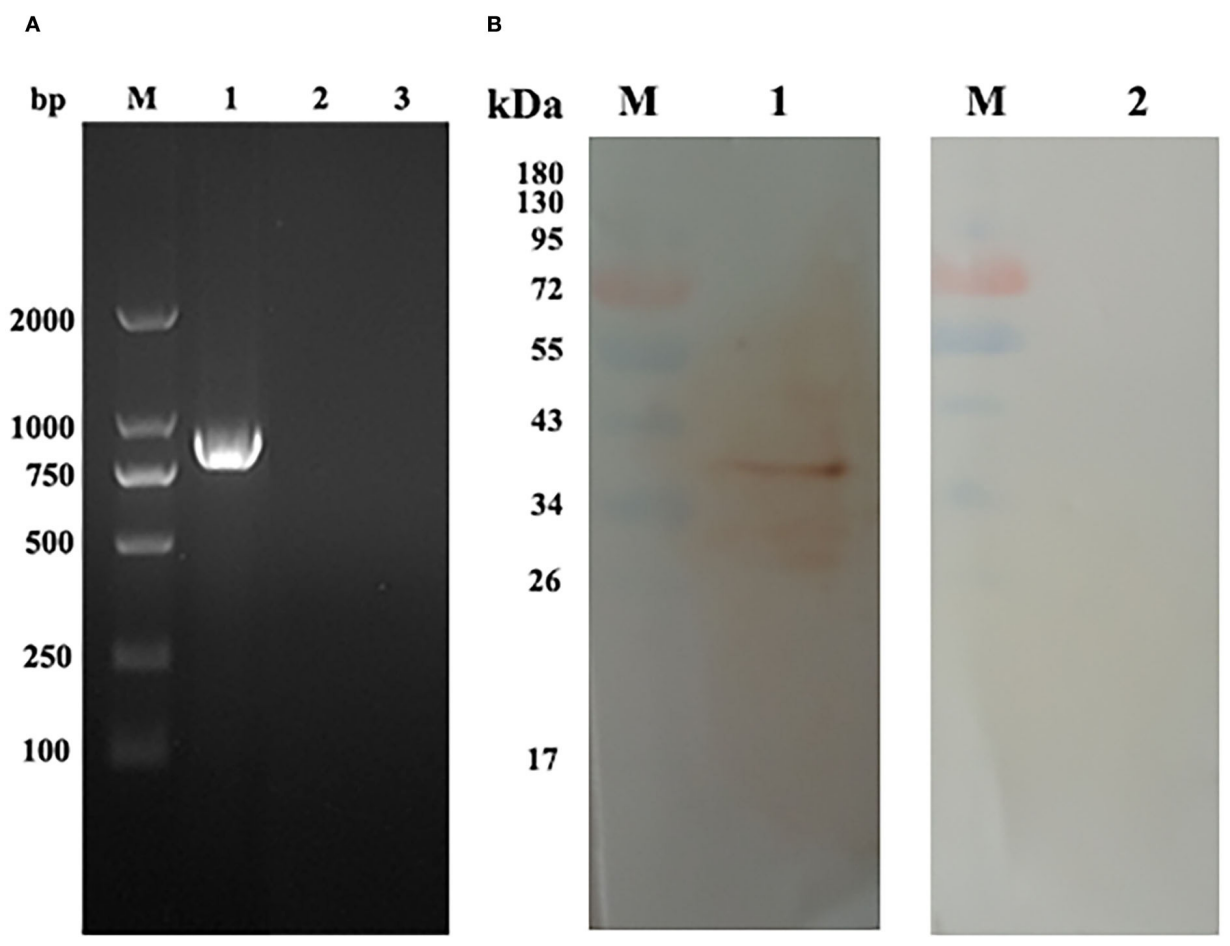
Transcription and expression of pEmROM1 in the injected muscles in chickens. **(A)** Transcription detection of pEmROM1 in chickens by RT-PCR. M: DNA molecular weight standard DL 2000. Lane 1: PCR product of EmROM1 detected from the pEmROM1 injected muscles. Lane 2: Detection of pVAX1.0 vector injected muscles. Lane 3: Detection of non-injection sites. **(B)** Expression detection of pEmROM1 in chickens by Western blotting. M: protein standard molecular weight. Lane 1: Detection of EmROM1 from the pEmROM1 injection site by anti-rEmROM1 rat serum. Lane 2: Negative rat serum control.

RT-PCR and Western blot assays were used to detect the transcription and expression of pEmROM1 at the injection site, respectively. A band of the same size as EmROM1 was detected from muscle tissues of immunized birds by RT-PCR, whereas no band was observed in the control groups (non-injection site and pVAX1.0 immunization) (Figure 3A). Western blot results showed that a single band slightly larger than the predicted molecular weight was identified by rat antiserum against rEmROM1 (Figure 3B). However, no band was identified by native rat serum. These results indicated that pEmROM1 at the injection site was transcribed and translated successfully in chickens.

### 3.4. EmROM1-induced immune responses in chickens

#### 3.4.1. EmROM1 elicited a significant cellular immune response in chickens

**Figure 4:**
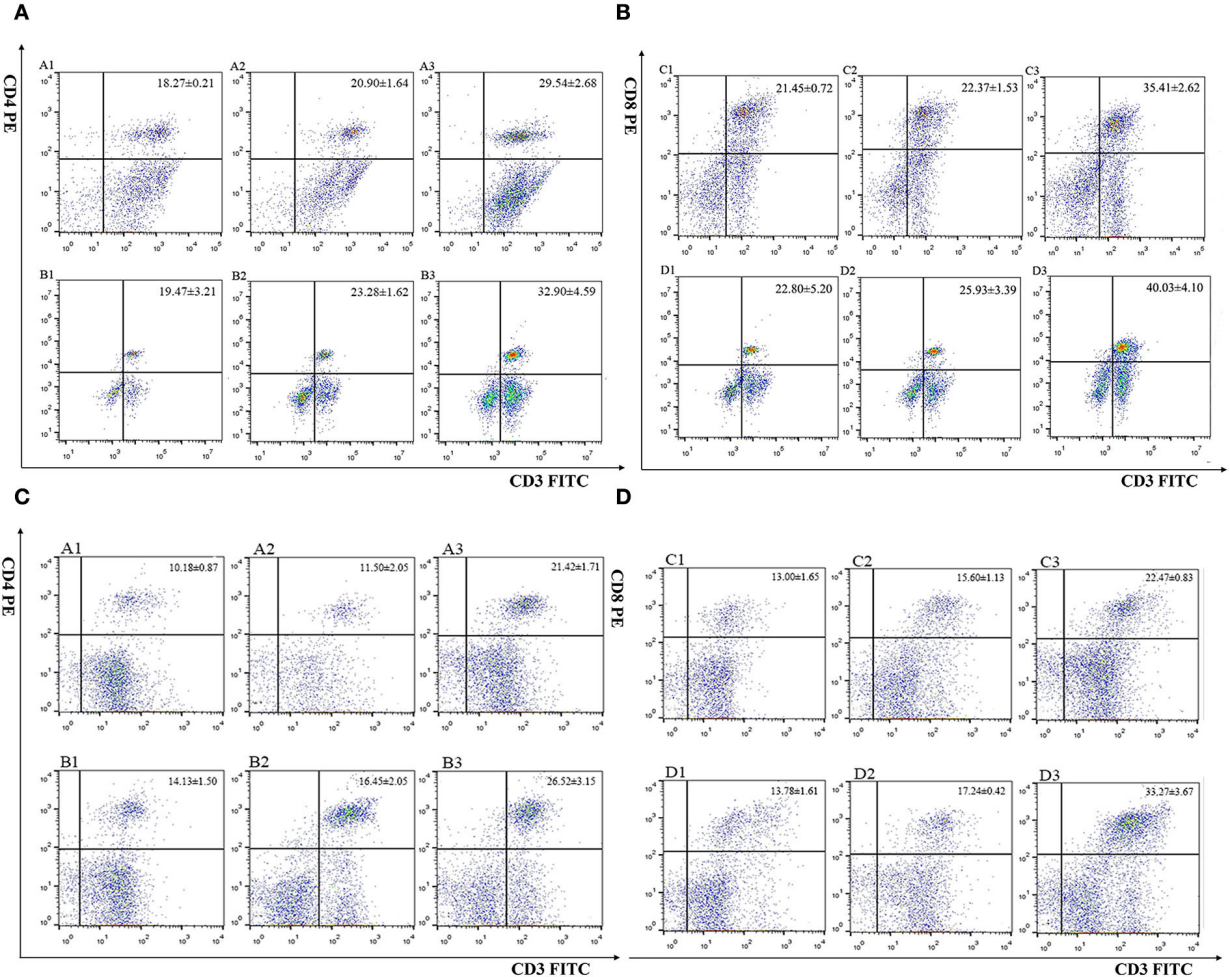
Flow cytometric analysis of splenic CD4^+^/CD3^+^ and CD8^+^/CD3^+^ T lymphocytes. **(A, B)** Percentage of T cell subpopulation in the spleen of rEmROM1 immunized chickens. **(C, D)** Percentage of T cell subpopulation in the spleen of pEmROM1 immunized chickens. **(A)** Quantification of CD4^+^/CD3^+^ lymphocytes in the chicken spleen at 21 days old (7 days after primary immunization). **(B)** Quantification of CD4^+^/CD3^+^ lymphocytes in the chicken spleen at 28 days old (7 days after booster immunization). **(C)** Quantification of CD8^+^/CD3^+^ lymphocytes in chicken spleen at 21 days old (7 days after primary immunization). **(D)** Quantification of CD8^+^/CD3^+^ lymphocytes in the chicken spleen at 28 days old (7 days after booster immunization). 1: PBS (negative control). 2: Quantification of T lymphocytes with immunized pET-32a tag protein or pVAX1.0 plasmid. 3: Quantification of T lymphocytes with immunized rEmROM1 or pEmROM1.

The percentage of CD4^+^ and CD8^+^ T cells in immunized chickens was examined by flow cytometry (Figure 4 and Table 1). The result revealed that there were no significant differences in CD4^+^ and CD8^+^ T cell percentage from the controls (PBS, pET-32a tag protein, and pVAX1.0 plasmid groups) 7 days after the primary and booster immunization (*P* > 0.05). Compared with the controls, the percentages of CD4^+^ and CD8^+^ T cells in rEmROM1-immunized and pEmROM1-immunized groups were significantly increased 7 days after the primary and booster immunization (*P* < 0.05).

**Table 1:** The proportion of T cell subpopulation in the spleen of immunized chickens (*n* = 5, value = Mean ± SD).

**Marker**	**Groups**	**1st** **immunization**	**2nd** **immunization**
CD4^+^/CD3^+^	PBS buffer	18.27 ± 0.21^a^	19.47 ± 3.21^a^
	pET-32a tag protein	20.90 ± 1.64^a^	23.28 ± 1.62^a^
	rEmROM1	29.54 ± 2.68^b^	32.90 ± 4.59^b^
	PBS buffer	10.18 ± 0.87^a^	14.13 ± 1.50^a^
	pVAX1.0	11.50 ± 2.05^a^	16.45 ± 2.05^a^
	pEmROM1	21.42 ± 1.71^b^	26.52 ± 3.15^b^
CD8^+^/CD3^+^	PBS buffer	21.45 ± 0.72^a^	22.80 ± 5.20^a^
	pET-32a tag protein	22.37 ± 1.53^a^	25.93 ± 3.39^a^
	rEmROM1	35.41 ± 2.62^b^	40.03 ± 4.10^b^
	PBS buffer	13.00 ± 1.65^a^	13.78 ± 1.61^a^
	pVAX1.0	15.60 ± 1.13^b^	17.24 ± 0.42^a^
	pEmROM1	22.47 ± 0.83^c^	33.27 ± 3.67^b^

a−c Means in the same columns marked with the same letter indicate that the difference between treatments is not significant (*P* > 0.05). Means in the same columns marked with a different letter indicate a significant difference between treatments (*P* < 0.05).

**Figure 5:**
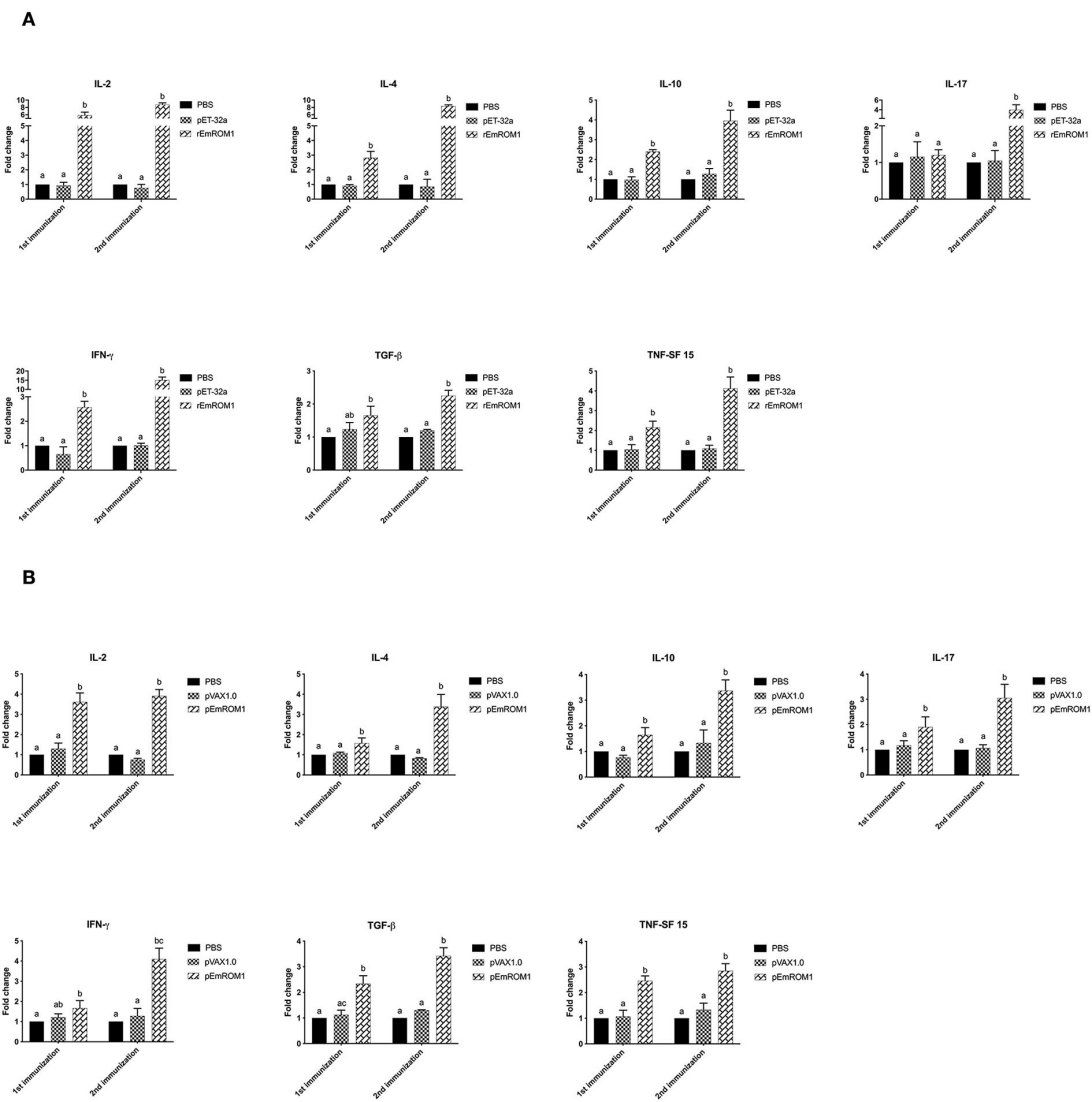
Transcription level of cytokines in sera from chickens immunized with rEmROM1 or pEmROM1. **(A)** Transcription level of cytokines in sera from chickens immunized with rEmROM1. **(B)** Transcription level of cytokines in sera from chickens immunized with pEmROM1. The error bars is regarded as the standard deviation, and five samples in duplicate respectively. Significant difference (*P* < 0.05) between groups is marked with different letters. No significant difference (*P* > 0.05) between groups is marked with the same letter.

The cytokine transcription levels in T lymphocytes were analyzed by qPCR. There were no significant differences from the controls (PBS and pET-32a tag protein groups) 7 days after the primary and booster immunization (*P* > 0.05; Figure 5A). Compared with the controls, the cytokine transcription levels from rEmROM1-immunized groups were significantly improved 7 days after the primary immunization (*P* < 0.05). However, there was no significant difference in the transcription level of IL-17 from the rEmROM1-immunized group compared with the controls (*P* > 0.05). Seven days after the booster immunization, the cytokine transcription levels except for IL-17 in the rEmROM1-immunized group were significantly increased than the controls (*P* < 0.05). Similar results were observed in the pEmROM1-immunized group (Figure 5B), and the cytokine transcription levels were significantly increased by immunization with pEmROM1 compared with the controls 7 days after the primary and booster immunization (*P* < 0.05). No significant differences were observed in the controls (PBS and pVAX1.0 plasmid groups) 7 days after the primary and booster immunization (*P* > 0.05).

#### 3.4.2. EmROM1 elicited a significant serum antibody response in chickens

**Figure 6:**
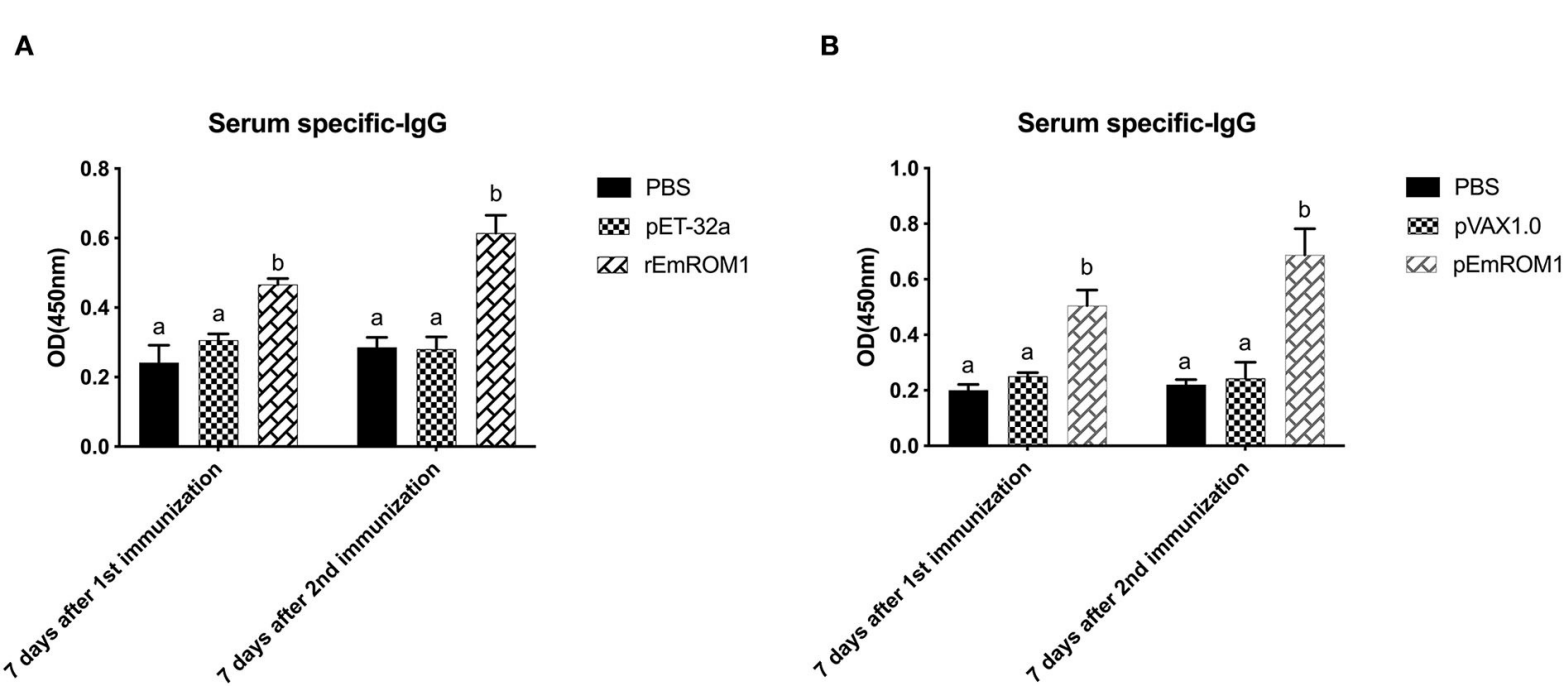
Serum specific IgG after immunized with rEmROM1 or pEmROM1. **(A)** Serum IgG titers induced by rEmROM1. **(B)** Serum IgG titers induced by pEmROM1. *N* = 20, the error bars = standard deviation. Significant difference (*P* < 0.05) between groups is marked with different letters. No significant difference (*P* > 0.05) between groups is marked with the same letter.

ELISA was used to detect EmROM1-induced specific IgG levels in chickens. The results showed that there was no significant difference in the controls (PBS, pET-32a tag protein, and pVAX1.0 plasmid groups) (*P* > 0.05). Compared with the controls, specific IgG levels in rEmROM1-immunized and pEmROM1-immunized groups were significantly increased 7 days after the primary and booster immunization (*P* < 0.05). The IgG levels were significantly prompted by booster immunization (Figure 6).

### 3.5. Assessment of protective efficacy induced by EmROM1

To evaluate the protective efficacy of EmROM1, two animal trials were carried out by immunization with rEmROM1 or pEmROM1 through *E. maxima* challenge in chickens (Table 2). Trial 1 shows the protective efficacy of rEmROM1. Weight gain in the rEmROM-immunized group was significantly improved compared with the control groups (challenged, pET-32a tag protein groups) (*P* < 0.05). Meanwhile, the immunization significantly decreased oocyst production and alleviated intestinal lesions compared with the controls (*P* < 0.05). As a result, immunization with rEmROM1 resulted in an ACI of 174.11, indicating moderate protection against *E. maxima*. The protective efficacy of pEmROM1 was assessed in trial 2. The immunization significantly improved weight gain and enteric lesions and decreased oocyst output of the pEmROM1 group compared with the control groups (challenged, pVAX1.0 plasmid groups) (*P* < 0.05). Immunization with pEmROM1 also produced an ACI of 163.37, indicating partial protection against *E. maxima*.

**Table 2:** Protective efficacy of rEmROM1 and pEmROM1 against *E. maxima* challenge (*n* = 30, value = Mean ± SD).

**Trials**	**Groups**	**Average body weight gain(g)**	**Relative body weight gain(%)**	**Mean lesion score**	**Average OPG (× 10^5^)**	**ACI**
1	Unchallenged control	56.91 ± 10.24^a^	100^a^	0 ± 0^a^	0 ± 0^a^	200
	Challenged control	27.21 ± 8.52^c^	47.81^c^	2.84 ± 0.88^c^	2.25 ± 0.94^c^	79.41
	pET-32a tag protein control	29.46 ± 11.25^c^	51.77^c^	2.66 ± 0.93^c^	2.15 ± 0.97^c^	85.17
	rEmROM1	51.68 ± 15.75^b^	90.81^b^	1.57 ± 0.65^b^	0.47 ± 0.40^b^	174.11
2	Unchallenged control	79.32 ± 9.59^a^	100^a^	0 ± 0^a^	0 ± 0^a^	200
	Challenged control	39.28 ± 9.72^c^	49.53^c^	2.83 ± 0.72^c^	2.81 ± 0.13^c^	81.23
	pVAX1.0 control	38.19 ± 15.39^c^	48.15^c^	2.75 ± 0.62^c^	2.80 ± 0.16^c^	80.65
	pEmROM1	63.19 ± 10.82^b^	79.67^b^	1.53 ± 0.71^b^	0.69 ± 0.15^b^	163.37

Trial 1 and Trial 2 were conducted separately, and the significance of data between these 2 trials was incomparable. The same letter (a–c) in the same columns indicates no significant difference between data in the same trial (*P* > 0.05). The different letter (a–c) in the same columns indicates a significant difference between data in the same trial (*P* < 0.05).

## 4. Discussion

ROMs have been demonstrated to be crucial for the invasion of some apicomplexan parasites due to their ability to cleave adhesins from the surface of the parasites, allowing them entry into the host cell completely (32–36). For instance, it has been confirmed that TgMIC2, TgMIC6, TgMIC12, and TgAMA1 were hydrolyzed by ROMs during the invasion of *T. gondii*, and PfEBA-175, PfAMA1, PfRh1, PfRh4, and PfTRAP was hydrolyzed by ROMs during the invasion of *Plasmodium falciparu*m (32). During the invasion of *E. tenella*, EtMIC4 may be hydrolyzed by EtROM3 (37). Therefore, ROMs were recently considered as potential vaccine candidates against infections by apicomplexan parasites. For example, it has been reported that DNA vaccination with ROM1, ROM4, and ROM5 provided appropriate protective efficacy against *T. gondii* infection (38–42). In *Cryptosporidium baileyi*, Yang et al. reported that vaccination with a DNA vaccine of pEGFP-CbROM reduced fecal oocyst burden (71.3%) in chickens after infection by *C. baileyi* (43). In *Eimeria*, ROMs have been reported to provide partial protection against homologous infection in the form of DNA vaccine, recombinant subunit vaccine, or recombinant BCG vaccine (22, 39, 43–47). These studies are consistent with our findings. In the present study, we found that EmROM1 elicited partial protection against *E. maxima* infection either in the form of recombinant protein or eukaryotic plasmid. Thus, our study provided an effective candidate antigen, EmROM1, for developing subunit vaccine and DNA vaccine against *E. maxima*. To some extent, it also indicates that EtROMs might be involved in the invasion process of the parasite, which needs to be further validated.

It is known that the cellular immune response plays a crucial role in *Eimeria* infection (5). In this study, EmROM1 immunization significantly increased the cytokine transcription levels and the percentages of CD4^+^/CD8^+^ T cells in vaccinated groups, which indicates that EmROM1 vaccination induced significant cellular immune responses. Our results are consistent with the previous reports (20, 22, 43, 47–49). Li et al. found that the cytokine transcription levels of IL-2 and IFN-γ and percentages of CD4^+^/CD8^+^ T cells were significantly increased in the recombinant protein ETRHO1 immunization group (20). Liu et al. showed that the percentages of several cytokines, such as IL-2 and IFN-γ, and CD4^+^/CD8^+^ T cell percentages increased significantly in the DNA vaccine group immunized with pVAX1.0-Rho vectors (47). Qi et al. (48) found that immunization with EtMIC1 could augment the transcription levels of IL-2 and IFN-γ in challenged birds. The role of the humoral immune response in the fight against *Eimeria* infection is controversial. Early studies believed that the ability of antibodies to resist *Eimeria* infection was minimal due to the observation that chickens bursectomized by hormonal and chemical means were resistant to *Eimeria* reinfection (50). By contrast, recent studies have shown that antibodies in protective immunity produced a marked effect against *Eimeria* because of their ability to prevent parasite invasion, development, and transmission and to provide passive and maternal immunity against infection (51). In this study, EmROM1 significantly increased the specific IgG levels after primary and booster immunization in the immunized chickens. These results might support the view that the antibody plays a role in the anticoccidial immune response. However, it needs to be further validated.

In this study, the intramuscular injection was used to evaluate the protective efficacy of EmROM1 due to its wide usage in vaccination. However, farmers do not prefer the intramuscular route because it is time-consuming and labor-intensive to administer, and it can cause stress to the animals, thus reducing their performance. Some non-injection routes are clinically popular, such as oral administration and spray. Therefore, the non-injection route could be used to evaluate the protective efficacy of EmROM1 to make it more promising for clinical application (52, 53). Theoretically, the DNA vaccine is immunized once, and the organism is able to produce the target antigen continuously (52). However, to obtain the best immune protection from the vaccines, both subunit and DNA vaccines were immunized twice in this study. In clinical, a single immunization is readily accepted by farmers given the cost and side effects of immunization on the animal. Therefore, the protective efficacy of a single immunization with this antigen can be evaluated in the future (54).

## 5. Conclusion

We evaluated the immune response and protective efficacy induced by EmROM1 in the forms of DNA and subunit vaccines. We found that EmROM1 vaccination could induce significant cellular and humoral immune response and provide partial protection against *E. maxima* challenge infection. Our results indicate that EmROM1 is a promising candidate antigen for the development of DNA vaccines or subunit vaccines against avian coccidiosis.

## Data availability statement

The original contributions presented in the study are included in the article/supplementary material, further inquiries can be directed to the corresponding author.

## Ethics statement

The animal study was reviewed and approved by the Committee on Experimental Animal Welfare and Ethics of Nanjing Agricultural University.

## Author contributions

XS conceived and designed this study. DT performed the experiments. LX and ML analyzed the data. MW drafted the manuscript. XL and RY helped to revise the manuscript. All authors contributed to the article and approved the submitted version.
